# Implications of within-farm transmission for network dynamics: Consequences for the spread of avian influenza

**DOI:** 10.1016/j.epidem.2013.03.001

**Published:** 2013-06

**Authors:** Sema Nickbakhsh, Louise Matthews, Jennifer E. Dent, Giles T. Innocent, Mark E. Arnold, Stuart W.J. Reid, Rowland R. Kao

**Affiliations:** a Institute of Biodiversity, Animal Health and Comparative Medicine, University of Glasgow, Bearsden Road, G61 1QH, Scotland, UK; b Population Health Research Centre, Division of Population Health Sciences and Education, St. George's University of London, Cranmer Terrace, London SW17 0RE, UK; c Biomathematics & Statistics Scotland (BioSS), The King's Buildings, Edinburgh, EH9 3JZ, UK; d AHVLA Sutton Bonington, The Elms, College Road, Sutton Bonington, Loughborough, LE12 5RB, UK; e Royal Veterinary College, University of London, Hawkshead Lane, North Mimms, Hatfield, Hertforshire, AL9 7TA, UK

**Keywords:** Mathematical modelling, Social network data, Poultry

## Abstract

The importance of considering coupled interactions across multiple population scales has not previously been studied for highly pathogenic avian influenza (HPAI) in the British commercial poultry industry. By simulating the within-flock transmission of HPAI using a deterministic S-E-I-R model, and by incorporating an additional environmental class representing infectious faeces, we tracked the build-up of infectious faeces within a poultry house over time. A measure of the transmission risk (*TR*) was computed for each farm by linking the amount of infectious faeces present each day of an outbreak with data describing the daily on-farm visit schedules for a major British catching company. Larger flocks tended to have greater levels of these catching-team visits. However, where density-dependent contact was assumed, faster outbreak detection (according to an assumed mortality threshold) led to a decreased opportunity for catching-team visits to coincide with an outbreak. For this reason, maximum *TR*-levels were found for mid-range flock sizes (~25,000–35,000 birds). When assessing all factors simultaneously using multivariable linear regression on the simulated outputs, those related to the pattern of catching-team visits had the largest effect on *TR*, with the most important movement-related factor depending on the mode of transmission. Using social network analysis on a further database to inform a measure of between-farm connectivity, we identified a large fraction of farms (28%) that had both a high *TR* and a high potential impact at the between farm level. Our results have counter-intuitive implications for between-farm spread that could not be predicted based on flock size alone, and together with further knowledge of the relative importance of transmission risk and impact, could have implications for improved targeting of control measures.

## Highlights

• Cross-scale dynamics were investigated for avian influenza in British poultry.
• Transmission risk is dependent on the assumed within-flock transmission mode.
• Transmission risk may not scale with transmissibility or flock size.
• Transmission risk corresponds with between-farm impact for 28% of farms.
• These results have implications for targeted disease control at the farm-level.

## Introduction

In social network analysis, when applied to epidemiology, it has typically been assumed that links between network nodes are static and therefore represent fixed, persistent contacts during an individual's infectious period (Huerta and Tsimring, 2002; Keeling, 1999; Meyers et al., 2005). Comparatively few models have allowed links to be dynamic – reflecting that, in reality, an individual's contacts are likely to change over time (Eames and Keeling, 2004; Kao et al., 2007; Volz and Meyers, 2009) and that links really represent the existence of the possibility of one or more ‘impulses’ whereby the infectious agent is passed from node to node. The dynamics of infection at the node-level has largely been a separate line of research (Gross and Blasius, 2008). In the few instances where cross-scale dynamics (i.e. combining the dynamics of the nodes and network structure) have been considered, they have revealed impacts on invasion and persistence thresholds that have important implications for disease control (Gross et al., 2006; Hufnagel et al., 2004).

Within the context of livestock diseases, simple models of static networks have assessed the benefit of including ‘transmission networks’ defined over the infectious period of the disease in question, and considering only network links thinned in inverse proportion to the probability that they are infectious (Kao et al., 2006), whilst more recent analyses have focused in more detail on the network dynamics (Bajardi et al., 2011; Vernon and Keeling, 2009). The impact of “super-shedding” cattle on persistence provides one example where the role of within-herd (node-level) dynamics in population transmission has been studied (Liu et al., 2007). In general, however, relatively little attention has been given to the interaction between node-level dynamics and network links.

Whether it is important to consider these factors in unison depends on the relative rates of change at the farm and network levels (Kao et al., 2007; Ochab and Gora, 2011; Volz and Meyers, 2009). For diseases such as highly pathogenic avian influenza (HPAI), which spreads rapidly at the farm level (i.e. node dynamics) (Bos et al., 2010; Elbers et al., 2004; Yoon et al., 2005), the opportunity for onward spread via epidemiologically relevant industry movements will depend on the timing of these movements.

HPAI is a public health threat with the potential to cause large economic losses; therefore, all countries require good contingency plans to limit the impact of outbreaks in commercial poultry. As the control of HPAI hinges on rapid detection and notification, improved understanding of within-flock transmission dynamics could help design measures to reduce transmission from infected premises. These dynamics are known to be influenced by management factors, such as flock size, and influence the time-to-detection of an outbreak (Savill et al., 2008). Although the implications of the within-farm transmission of HPAI for indirect farm-to-farm transmission via movements of people, vehicles and equipment has been considered (Dorea et al., 2010), previous studies have not considered in detail the dynamics of within-flock transmission (such as the impact of flock size and assumed transmission mode) or the impact of the timing of on-farm movement activities.

Catching team personnel (through clothing and equipment), forklift trucks, and slaughterhouse vehicles are considered to be important risk factors for the spread of disease within the British commercial poultry industry (Anon, 2006; Gittins and Canning, 2006). Using temporally explicit data describing the daily on-farm visit schedules for a major poultry catching company located in England (Dent et al., 2011), we explored the interaction between the within-flock transmission dynamics of HPAI, measured in continuous time, and temporally explicit catching-team visits, measured in discrete time. These on-farm visits made by catching teams represent the risk of fomites (i.e. people, vehicles or equipment) being exposed to HPAI and subsequently spreading infection through their movements, and not the movement of the birds themselves.

We present an explicit exploration of the interaction across these time-scales by combining a deterministic model of disease spread (given the typically large flock sizes for commercial poultry) with the empirical data describing on-farm catching-team visits. As these on-farm visits represent ‘impulses’ enabling the spread of HPAI between-farms, we gain insights that have implications for the dynamics of network links between premises. Our results highlight the impact of the characteristics of bird-to-bird transmission on the potential for spread from infected premises. Additionally, we identify the farm-level factors that are predictive of high relative transmission risk (see below) and discuss how this can be used to inform the risk of HPAI spread between farms.

## Methods

### Within-flock transmission

A S-E-I-R model was used to track the number of birds in susceptible (S), exposed (E), infectious (I) and removed (R) classes. The ‘R’ class represents the end of a birds’ infectious period as a result of HPAI-induced mortality and therefore these birds no longer contribute to the force of infection within the flock. In addition, a ‘F’ class was incorporated to represent the environmental build-up of infectious faecal material within the poultry house. This ‘F’ class determined the potential for the exposure of fomites such as catching team personnel, forklift trucks, slaughterhouse vehicles and equipment. In order to gain a better understanding of the relationships amongst several flock-level factors under different transmission mode assumptions, we captured average effects using a deterministic framework and thus do not account for stochastic variability in the within-farm dynamics. However, we do consider uncertainty and between farm variability in the parameter values of this model (see below).

The within-flock model was developed to reflect floor-reared broiler poultry, which was the predominant production type captured by our catching-company movement data. These data reflect the daily schedules of the catching teams that are responsible for transporting poultry to the slaughterhouse vehicle; in the case of broiler chicken production, this either relates to flock thinning, partial bird depopulation or total bird depopulation at the end of the production cycle.

Each bird was assumed to excrete *ε* grams of faeces per hour; *εI* therefore gave the quantity of infectious faeces excreted by the flock each hour. The amount of infectious faecal material was assumed to decay at 5% per hour, consistent with available experimental data (Shortridge et al., 1998) and a published within-flock model for HPAI in poultry (Savill et al., 2006, 2008).

As the relative contribution of different transmission mechanisms is poorly understood (Spekreijse et al., 2011a), the total infection pressure, *β*, was modelled as the sum of the infection pressure due to two independent routes; namely transmission via: (i) aerosol (*β*_*a*_), which represented direct bird-to-bird contact (i.e. dependent on ‘*I*’); and (ii) via infectious faeces (*β*_*f*_), which represented indirect contact via faecal contamination of dust and drinking/feeding equipment (i.e. dependent on ‘*F*’). As the mechanisms of bird-to-bird contact (direct or indirect) and the likely rates of contact are poorly understood, both frequency-dependent (FD) and density-dependent (DD) modes of transmission were considered. For FD transmission the ODEs are given by

$$\begin{array}{l} \frac{dS}{dt}=-S\left(\beta_{a}\frac{I}{N}+\beta_{f}\frac{F}{N}\right),\\ \frac{dE}{dt}=S\left(\beta_{a}\frac{I}{N}+\beta_{f}\frac{F}{N}\right)-\delta E,\\ \frac{dI}{dt}=\delta E-\gamma I,\\ \frac{dR}{dt}=\gamma I,\\ \frac{dF}{dt}=\epsilon I-\sigma F \end{array}$$

The expressions for DD transmission are identical except that the force of infection is replaced with

$$\frac{\beta_{a}}{\bar{n}}I+\frac{\beta_{f}}{\bar{n}}F\text{,}$$

where the transmission rates are scaled by the mean flock size $\left(\bar{n}\right)$ to facilitate comparison with the FD models. Table 1 contains a full list of parameter values and ranges.

We chose a range of values for the basic reproduction number, *R*_0_, from 2 to 38 (corresponding to *β* values ranging 0.01–10 h^−1^) to explore a spectrum of scenarios that are covered by experimental and modelling studies. For example, estimates of *R*_0_ are as low as 1.2 and 2.2 in experimental transmission studies and field data respectively (Bouma et al., 2009; Spekreijse et al., 2011b; Tiensin et al., 2007), while estimates as high as 22–66 have been assumed in mathematical models due to the greater flock size and infection pressure expected within a commercial flock (Savill et al., 2006; Sharkey et al., 2008; Truscott et al., 2007). Supplementary Material Section 1 includes details of model sensitivity to the *R*_0_ range.

*R*_0_ was calculated as the dominant eigenvalue of the Next Generation Matrix, *ρ*(*M*), where

$$M=\left(\begin{array}{ccc} 0 & x_{a}\cdot \frac{1}{\gamma } & x_{f}\cdot \frac{1}{\sigma }\\ 1 & 0 & 0\\ 0 & \frac{\epsilon }{\gamma } & 0 \end{array}\right)\text{,}$$

and where *x*_*a*_ = *β*_*a*_/*N* and *x*_*f*_ = *β*_*f*_/*N* for FD transmission, and $x_{a}=\beta_{a}/\bar{n}$ and $x_{f}=\beta_{f}/\bar{n}$ for DD transmission.

The average flock size per farm (*N*, the ratio of number of poultry to number of poultry houses) was divided into quintiles, using the distribution obtained from the Catching Company Database (mean = 22,465 birds, range = 4571–45,667 birds). Twenty values each of *β*_*a*_ and *β*_*f*_ were chosen to cover the possible range likely on farms (0.01–10 h^−1^). All flock size and *β* combinations (400 pairwise combinations of *β*_*a*_ and *β*_*f*_) were run for both FD and DD transmission modes and for each quintile. In total, 4000 outbreaks (400 *β* combinations × 5 flock sizes × 2 transmission scenarios) were simulated using Matlab v.7.8.0 (The MathWorks, Inc., Natick, MA, USA). Each simulation represented transmission within a single flock and one single infected bird initiated each outbreak at time zero.

### Relative transmission risk

Model simulations were used to explore the effect of within-flock transmission characteristics on a farms’ relative transmission risk (*TR*) (i.e. relative across farms). Empirical data was obtained from a major catching company in England which provided the daily schedules of catching-teams recorded on an hourly basis. This Catching Company Database represented 68 catching teams visiting a total of 415 farms over a total period of 950 days (see Dent et al., 2011 and Supplementary Material Section 2). Each farms’ recorded catching-team visit days were given a value determined by the total number of slaughterhouse vehicle loads (*φ*) associated. The *TR* of an infected farm was then computed by matching each of their daily on-farm visits to the corresponding day of all the simulated within-flock outbreaks that matched the relevant flock-size.

Specifically, for farm *i* given a specified day of incursion *z* (the day within the movement data that was identified as day zero of the simulated outbreak), the relative transmission risk *TR*_*i*,*z*_ was given by the product of the amount of infectious faecal material (*F*_*i*,*z*,*t*_) and the number of vehicle loads (*φ*_*i*,*z*,*t*_), summed over each day, *t*, of the outbreak:

$$TR_{i,z}=\sum_{t=1}^{t_{\max}}F_{i,z,t}\cdot \varphi_{i,z,t}$$

To account for the intra-farm heterogeneity in movement pattern (i.e. both frequency of catching-team visits and bird transportation capacity of slaughterhouse vehicles), iterations over all possible incursion days (*z*) were run. As a result of this intra-farm heterogeneity in the movement patterns, the number of incursion day iterations varied per farm. An overall relative transmission risk at the farm-level, *TR*_*i*,*sim*_, was calculated by averaging across all incursion day iterations (*z*):

$$TR_{i,sim}=\frac{\sum_{z=1}^{z_{\max}}TR_{i,z}}{n_{i}}\text{,}$$

where *n*_*i*_ represents the total number of iterations (*z*) corresponding to farm *i*. We consider two types of infection incursion. First, it was assumed that an incursion event would occur only on movement days, corresponding to farm-to-farm transmission via a catching team whose previous visit was to an infectious premises. Second, incursions could occur on any day thereby representing other sources of introductions such as a wildlife reservoir (for details see Supplementary Material Section 3).

It was assumed that once a threshold-level of dead birds (known as the mortality threshold, MT) had been reached, the outbreak would be detected and any further risk of transmission prevented. Following an outbreak of HPAI H7N7 in the Netherlands in 2003, a MT of 0.5% (of the initial susceptible flock) per day for two consecutive days was recommended for Dutch broiler producers (Elbers et al., 2007). However, MTs are recognised to vary according to several factors such as production type, management practices and bird age and a wider likely range of 0.03–3.33% was found for poultry producers in Georgia, USA (Vieira et al., 2009). As the likely MT triggering HPAI detection in British poultry farms is not known we considered a range of MTs and present results for an intermediate threshold of 0.5%, corresponding to the Dutch recommendation.

### Identifying predictors of high relative transmission risk

To identify factors that have the greatest effect on the probability of transmission from a farm, we produced a general linear regression model to compare the model inputs with the predicted relative transmission risk. To remove the effect of uncertainty in transmission parameters, the farm-level transmission risk $\left({\bar{TR}}_{i}\right)$ corresponding to a mid-range transmissibility scenario (*β*_*a*_ + *β*_*f*_ ~ 10) was chosen. We wished to identify the most important drivers of transmission risk, therefore only main effects in the statistical model were considered. For full details of predictor variables see Table 2.

A square root transformation was applied to ${\bar{TR}}_{i}$ as this improved the normality of residuals and decreased heteroscedasticity. The models were built using a backwards stepwise method and AIC to assess the fit at each stage in the model development. The inclusion of ‘company integration’ as a random effect was considered but not included in the final models due to the few companies with an adequate group size (only two companies had more than two associated farm premises).

The most influential data points, as identified by their Cook's statistic, were assessed for their impact on the model coefficients and their significance levels. To assess the overall effect of covariates in the model, the relative transmission risk was predicted using the linear model with all parameters set at the median (covariates) or modal (factors) value. The effect of varying a single covariate or factor across the range observed within the dataset was then computed. Those covariates or factors with the greatest effect were recorded. All statistical analyses were carried out in R software v.2.14.2.

### Correlation between relative transmission risk and network connectivity

To assess the potential impact on infection propagation between farms, ${\bar{TR}}_{i}$ was cross-classified with a measure of between-farm association frequency, informed by the Poultry Network Database (PND). This database consisted of surveys of: (i) single-site and (ii) multi-site farm premises, (iii) slaughterhouses and (iv) catching companies; these data were used to infer potential associations between farms arising through shared industry associations. For example, farms that used the same slaughterhouse, catching company or that were integrated as part of a larger company were assumed to be epidemiologically linked (Dent et al., 2008; Nickbakhsh et al., 2011).

Using these associations informed by the PND, a between-farm association matrix was generated for the farms captured by the Catching Company Database that were used in the computation of ${\bar{TR}}_{i}$ (*n* = 108). The total number of associations per farm was assumed to represent a maximum potential for propagation via the poultry network. In this way, correlation between the farm-level risk of exposing personnel, vehicles and equipment during farm visits (i.e. as measured through ${\bar{TR}}_{i}$), and the potential risk of propagation occurring through the industry network, was determined.

## Results

### Movement data

**Fig. 1:**
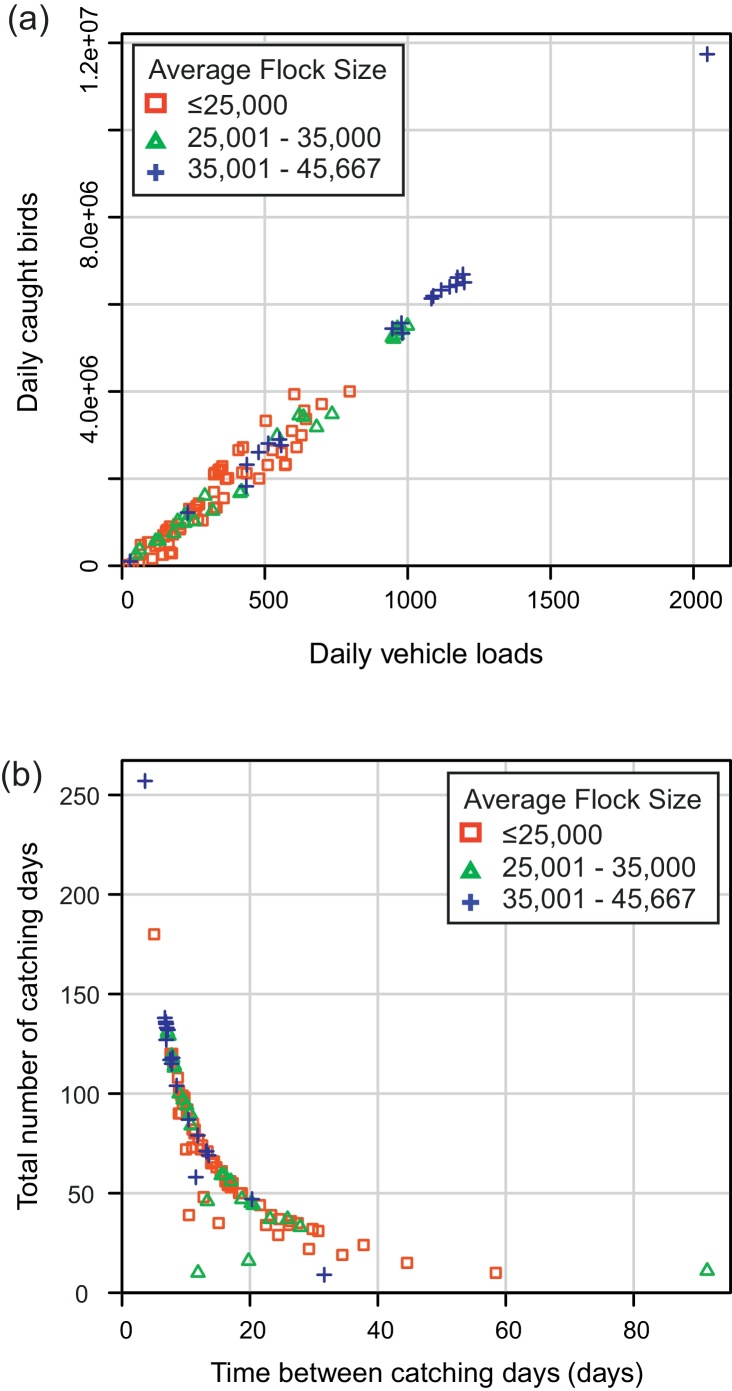
Characterising farms by their catching-team visits and average flock size. (a) The mean daily vehicle loads and mean daily caught birds have a linear relationship, and (b) the mean time (measured in days) between catching days declines exponentially with the mean total number of catching days.

A descriptive analysis of the input data showed that the farm-level mean number of caught birds and average flock size both increased linearly with the mean daily number of vehicle loads (Fig. 1a). In contrast, the mean total number of catching days (across the entire dataset) and flock size decreased with the mean time-interval between consecutive visit days, *T*_*b*_ (Fig. 1b). Overall, larger flocks tended to have a greater number of daily vehicle loads, a greater total number of visits, and a lower *T*_*b*_. Further inspection of *T*_*b*_ showed it to have a variance-to-mean ratio exceeding 1, indicating temporal clustering of visits. This temporal clustering at the farm-level reflects two important activities related to these catching teams – flock thinning mid-production cycle and partial depopulation at the end of the production cycle (see Supplementary Material Section 3 for an example data snapshot highlighting this temporal pattern of catching team visits).

**Fig. 2:**
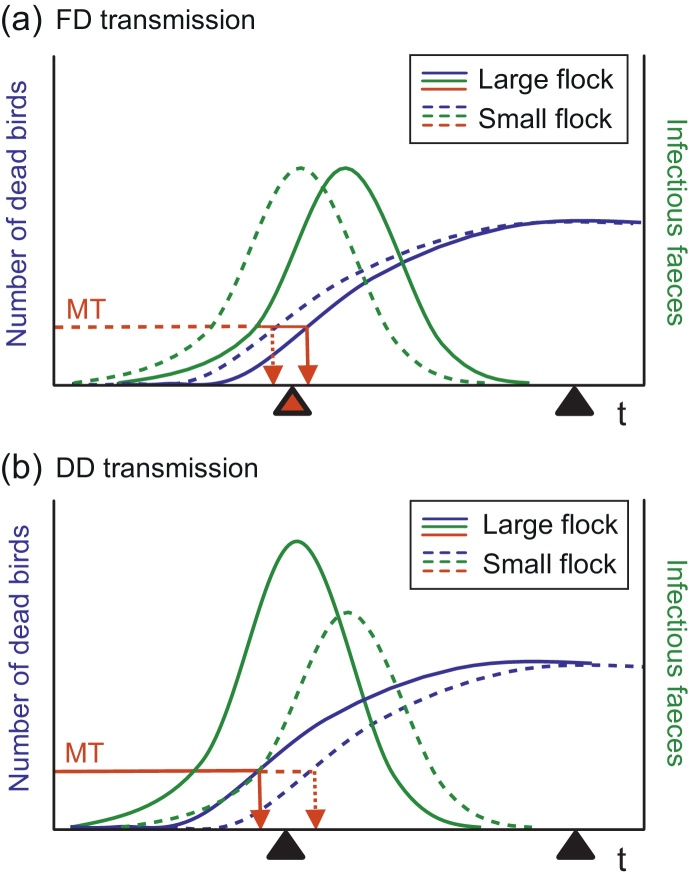
Schematic of the cross-scale interactions between within-flock transmission dynamics and the timing of catching-team visits. The within-flock dynamics corresponding with dead birds (i.e. the R or “removed” model class) and infectious faeces (i.e. the F or “infectious faeces” model class) follow blue and green curves respectively. The opportunity for a catching-team visit to coincide with the outbreak is shown for small (dotted lines) and large (solid lines) flock sizes under (a) frequency-dependent (FD) transmission, and (b) density-dependent (DD) transmission. Red lines indicate the number of dead birds corresponding with the mortality threshold (MT). Triangles demonstrate the case where increases to flock size results in catching-team visits coinciding with an outbreak (in red), and occurring outside the outbreak window (in black).

For FD transmission, the time-to-detection increased with increasing flock size (solid red arrow compared to dashed red arrow, Fig. 2a), as previously highlighted (Savill et al., 2008). However, for DD transmission, time-to-detection decreased with increasing flock size (solid red arrow compared to dashed red arrow, Fig. 2b). In this case, greater infection pressure and greater movement activity, but more rapid outbreak detection, interacts to influence the potential for transmission.

### Cross-scale interactions at the farm-level

The model simulations, combining the deterministic within-flock transmission model for HPAI with the catching-team movement data, showed that for a given flock size, transmission mode and mortality threshold, the average *TR* (across all farms) increased with transmissibility (approximately scaling with the build-up of infectious faeces), peaked at high transmissibilities (*R*_0_ ~25–30) and then dropped sharply for even higher transmissibilities – a consequence of the more rapid accumulation of dead birds. This occurred even for relatively small increases to *β*_*a*_ or *β*_*f*_ of around one additional transmission event per bird per day, up to a level which depended on the assumed transmission mode (discussed further below).

**Fig. 3:**
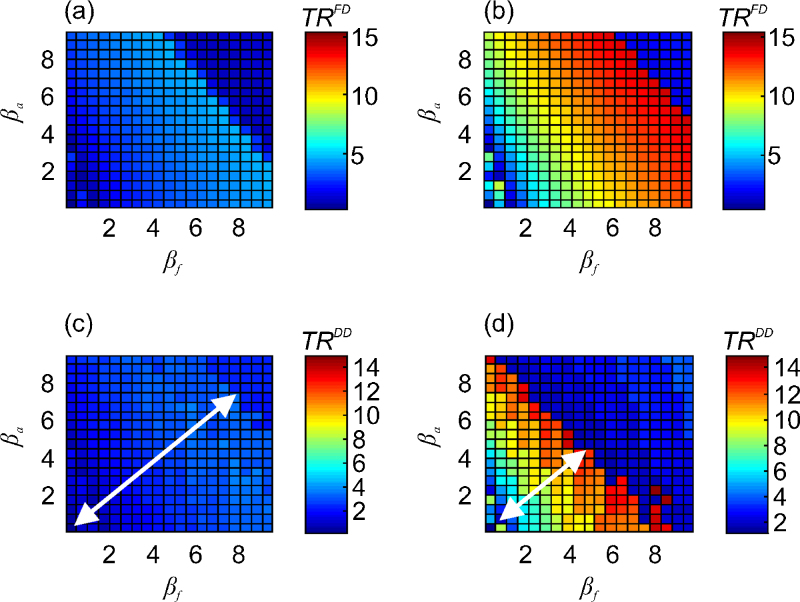
Relative transmission risks (*TRs*) for a range of transmissibility scenarios. Assuming frequency-dependent (FD) transmission (*TR*^*FD*^) for (a) small (~4500–25,000 birds) and (b) large (~35,000–45,600 birds) flocks, and assuming density-dependent (DD) transmission (*TR*^*DD*^) for (c) small (~4500–25,000 birds), and (d) large (~35,000–45,600 birds) flocks. The white arrows in (c) and (d) demonstrate the relative difference in the region of parameter space between the lowest and highest *TR* as flock size increases under DD transmission. These analyses assumed outbreak detection occurred when at least 0.5% mortality was reached for two consecutive days. All other parameters were set to default values (see Table 2).

For frequency-dependent (FD) transmission, an increase to flock size increased *TR* (Fig. 3a and b). However, for density-dependent (DD) transmission, although *TR* scaled with flock size for low-mid-range transmission rates (i.e. *β*_*a*_ + *β*_*f*_ < 10), for mid-to-high range transmission rates (i.e. *β*_*a*_ + *β*_*f*_ > 10) earlier disease detection caused *TR* to peak at lower transmission rates as flock size increased. This narrowing of the region of parameter space between the lowest and highest *TR* values with an increase to flock size is illustrated by the relative sizes of the white arrows in Fig. 3c and d. As the number of dead birds approached the mortality threshold, *TR* became highly sensitive to the balance between infectious faeces and time-to-detection under DD transmission (see bottom right red region of Fig. 3d); the greater environmental build-up of infectious faecal material counteracted the shortened time-to-detection for small regions of parameter space causing *TR* to oscillate as transmission parameters varied.

The results presented assume a mortality threshold of 0.5%. Given the likely variability and lack of recent experience of HPAI by farmers in Great Britain, we compare this to MTs of 0.3% and 0.7% (Supplementary Material Section 4). We note that the range of infection pressures corresponding with high *TR* increases with increasing MT, whilst the rapid drop in *TR* phenomenon occurs later.

### Generating farm-level profiles for relative transmission risk

Tables 3a and 3b show the multivariable model results under FD and DD transmission respectively. See Supplementary Material Section 5 for details of the multivariable model diagnostics. Overall, the time between consecutive catching-team visit days, *T*_*b*_, had the largest average effect on ${\bar{TR}}_{i}$ for both transmission modes (linear coefficients = −0.033, *p* ≤ 0.008; for both model scenarios). However, when considering the range of possible effect size across all farms for each predictor in the multivariable model, ‘mean daily vehicle loads’ had the potential to have the largest impact under FD transmission (see Supplementary Material Section 6).

**Fig. 4:**
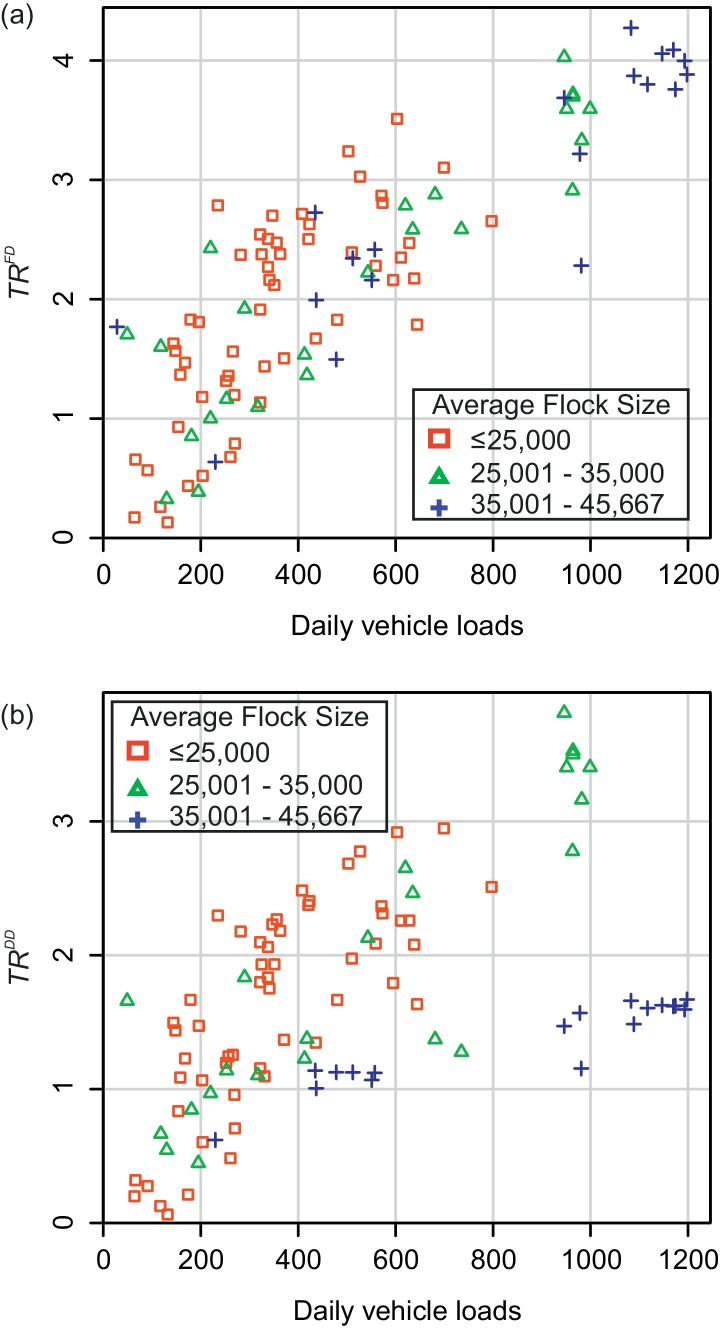
The effect of average flock size and mean daily vehicle loads on the relative transmission risk (a) assuming frequency-dependent (FD) transmission, and (b) assuming density-dependent (DD) transmission. See Table 2 for a full description of the model variables. The transmission risk corresponds with a mid-range level of transmissibility where *β*_*a*_ ~ 5 and *β*_*f*_ ~ 5.

The relationship of ${\bar{TR}}_{i}$ with flock size varied depending on the transmission mode, as shown in Fig. 4; a significant effect was found only in the univariable model under FD transmission (results not shown, linear coefficient = 4 × 10^−5^, 95% CI: 2 × 10^−5^ to 6 × 10^−5^, *p* < 0.0001), whilst under DD transmission the average flock size had a significant negative impact in the multivariable model, an effect which was further modified by ‘mean daily vehicle loads’ (interaction parameter between ‘flock size’ and ‘mean daily vehicle loads’ = −1.21 × 10^−7^, *p* < 0.0001). Therefore, for DD transmission, the relationship with ‘mean daily vehicle loads’ was seen most clearly for small and medium sized flocks, with *TR* peaking at mid-range flock sizes (~25,000–30,000 birds).

### Generating between-farm profiles for relative transmission risk

**Fig. 5:**
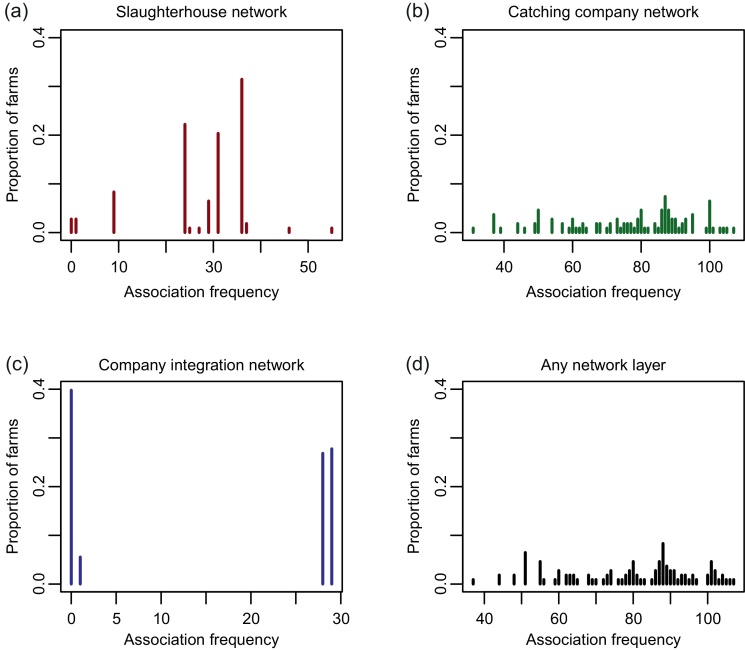
Between-farm association frequency distributions. Between-farm associations were assumed to occur through farms sharing (a) the same slaughterhouse, (b) catching company, (c) through company integration, or (d) through at least one of these network layers. These results were generated using a subset of farms for which estimates of transmission risk had been generated (*n* = 108 farms).

Fig. 5 shows the distribution of between-farm association frequencies for slaughterhouse, catching company, integrated company and combined layers of the poultry network, for all farms recorded in the Catching Company Database (*n* = 108). However, it should be noted that the median between-farm association frequency overall combining all network layers, when based on the full Poultry Network Database, was estimated to be 343 farms (range = 105–1453 farms).

**Fig. 6:**
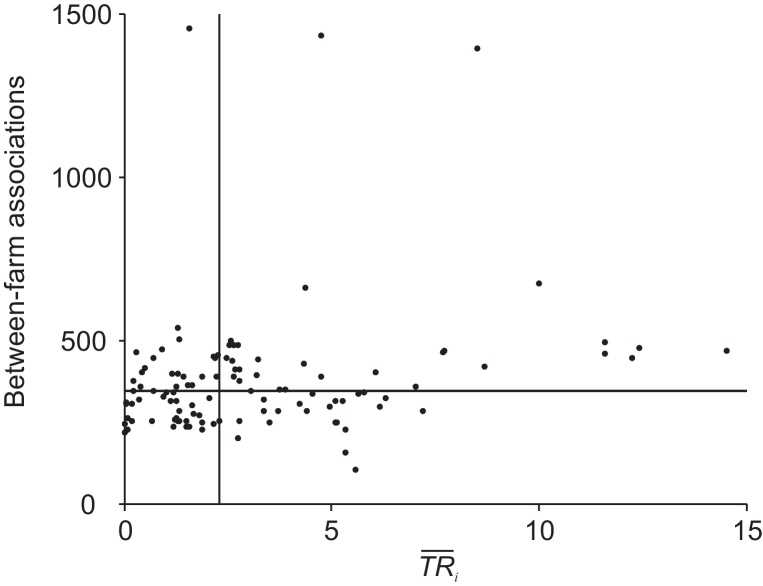
Cross classifying relative transmission risk with the between-farm association frequency. The total number of between-farm associations was used to indicate network connectivity via shared slaughterhouses, catching companies or through company integration, per farm. The horizontal line indicates the median between-farm association frequency; the vertical line indicates the median $\bar{TR}$. The transmission risk corresponds with a mid-range level of transmissibility where *β*_*a*_ ~ 5 and *β*_*f*_ ~ 5.

Farms with high estimates for both between-farm associations and ${\bar{TR}}_{i}$ would produce the highest risk of a widespread epidemic. When cross-classifying the between-farm association frequency with ${\bar{TR}}_{i}$, a large proportion of farms had a below median value for both factors (21% of farms, see bottom left quadrant of Fig. 6). However, a further 21% of farms had a relatively low estimate of ${\bar{TR}}_{i}$ and a relatively high estimate of between-farm association frequency (see top left quadrant of Fig. 6). The largest fraction of farms (28%) had the highest risk combination, with above median estimates for both factors (see top right quadrant of Fig. 6). No difference was found in the distribution of farms between FD and DD transmission modes when dichotomising ${\bar{TR}}_{i}$ by median values.

## Discussion

### Cross-population scale interactions

The integration of infection transmission dynamics at the within-group and population level is increasingly important as greater demands are made of predictive mathematical models (Haydon and Matthews, 2007; Kao et al., 2007). For pathogens that are likely to spread rapidly within a farm, the risk of onward transmission via fomites depends on the opportunity for an on-to-farm movement to coincide with an outbreak. We have explored the importance of these cross-scale interactions for highly pathogenic avian influenza (HPAI) within the British commercial broiler poultry industry. By considering the interaction between the farm-level infectious period and explicit temporal pattern of catching-team visits, our results have implications for the between-farm spread, or the network dynamics, of HPAI.

Poultry flock size may be indicative of both the time to detection of an outbreak, and the expected amount of on-to-farm movement activity. However, this does not account for the trade-off between the build-up of faecal virus within the poultry house and the opportunity for virus exposure. Our results suggest that the overall effect of flock size on the relative transmission risk (*TR*) is sensitive to the bird-to-bird transmission mode. For frequency-dependent (FD) transmission, the time-to-detection increased with flock size (Fig. 2a), as highlighted previously by Savill et al. (2008). However, for density-dependent (DD) transmission, greater infection pressure and greater movement activity, but more rapid outbreak detection, interacts to influence the opportunity for onward spread from larger flocks (Fig. 2b). This interaction had not previously been considered with respect to HPAI within commercial poultry.

As the infection pressure (i.e. *β*_*a*_ + *β*_*f*_) increased, so did the amount of infectious faeces thus increasing *TR*; this counteracted the shortened time-to-detection for DD transmission for some regions of transmissibility but *TR* was highly sensitive to the balance between these factors causing an oscillatory effect (Fig. 3d). Overall, mid-range sized flocks (~25,000–35,000 birds) were found to have the greatest *TR* under DD transmission, in contrast to larger flocks (~35,000–45,600 birds) under FD transmission.

Faeces are not removed mid production cycle in British commercial broiler chicken farms, therefore it was assumed that the *TR* depended on the product of available infectious faecal material and the number of vehicle loads. However, a nonlinear dependence (e.g. an upper contamination threshold) could change the sensitivity to flock size and the impact of transmission mode assumptions. Given the lack of empirical data available in relation to viral survival times we assumed a constant rate of viral decay in line with previous published studies (Savill et al., 2006, 2008). Whilst we cannot postulate the appropriateness of alternative assumptions, we anticipate that this would not impact on our qualitative results.

### Generating farm-level risk profiles

The identification of predominantly movement-related predictors that depend on the bird-to-bird transmission mode has important implications for disease control strategies. For example, the multivariable regression analyses suggest that reducing the overall frequency of on-to-farm visits would be more effective for FD transmission, in contrast to limiting the clustering of consecutive visit days for DD transmission (Tables 3a and 3b). As the temporal clustering of visits at the farm-level was strong, activities requiring on-to-farm visits mid-production cycle, such as the practice of flock-thinning, might be particularly detrimental for DD transmission characteristics. Such practices are recognised risk factors for disease transmission within the British poultry industry (ACMSF, 2005; Slader et al., 2002). An improved understanding of the likely bird-to-bird contact behaviour, and therefore the predominant mechanism of transmission within a poultry flock, is needed to better characterise this risk.

In our model, the highest potential for spread via catching-team visits was associated with outbreaks of HPAI virus with high transmissibility (*R*_0_ ~25–30), in large broiler flocks, under FD transmission. Delayed detection of the outbreak by a farmer (as demonstrated by increases to the mortality threshold triggering outbreak detection, see Supplementary Material Section 4) would further increase this risk. Delayed notification might be expected for less virulent poultry pathogens such as low pathogenic avian influenza (LPAI), which despite sometimes significant mortality (Bano et al., 2003), typically has less severe morbidity and lower mortality (Defra, 2006). This observation has important implications for the spread of HPAI, as the undetected circulation of LPAI virus precursors may increase the likelihood of a subsequent HPAI outbreak (Mannelli et al., 2006).

Though particular HPAI subtypes, such as H5N1, are expected to be detected relatively rapidly, with some analyses indicating no longer than one week (Yoon et al., 2005), evidence from experimental work using the H7N7 subtype, implicated in an outbreak in the Netherlands in 2003, suggests that HPAI may have been left to circulate within a flock undetected for 11–15 days (Bos et al., 2007). Due to the difficulties in directly extrapolating from experimental studies typically involving very few birds (i.e. typically a maximum of five susceptible in-contact birds, in the absence of concurrent infection and environmental factors that may enhance transmission under field conditions), the upper limit for transmission rates in relation to commercial poultry flocks may be greater than estimated by these studies.

In view of this likely variability between virus subtypes, and the possible under-estimation of transmission rates by experimental studies, we explored a range of time to detections (2–10 days), representing a range of plausible HPAI outbreak characteristics, reflecting in particular the most virulent HPAI viruses. Furthermore, in view of the impact of farmer behaviour, we also considered a range of mortality thresholds, MTs, and find that as the MT increases, *TR* increases for a greater range of infection pressures; for large flocks and thresholds beyond 0.7% we might not observe the “rapid drop in *TR*” phenomenon as the infection pressure increases within the range considered (see Supplementary Material Section 4).

### Generating between-farm risk profiles

If the most highly connected farms also have a high *TR*, the overall risk of a widespread outbreak could be high; this was the case for 28% of farms in these analyses. However, a large proportion of farms (42%) had discordant network-connectivity and relative transmission risk characteristics (Fig. 6); approximately half of these (21%) had a low estimate of *TR* (corresponding to a mid-range transmissibility scenario, ${\bar{TR}}_{i}$) but a high between-farm association frequency. In this case a high impact at the between-farm level but with a low associated risk of occurrence could be construed as representing a medium-level risk.

A more detailed understanding of the relative importance of farm-level transmission “risk” and between-farm “impact” would allow us to better characterise farms and determine the most appropriate control measures. For example, categorising farms based on combinations of exposure “risk” and overall “impact” may enable the effective targeting of control measures – via a relatively greater focus on preventive measures such as farm-level biosecurity for farms with high estimated transmission risk, and a relatively greater focus on contact-tracing during an outbreak for farms with high estimated between-farm association frequency. Farms with high estimates for both factors warrant the greatest focus from prevention and control measures.

The most crucial factor for minimising the risk of spread between farms will be the rapidity with which farmers notify and report a suspected outbreak, as discussed for farm-level risk profiles in relation to assumed mortality thresholds. In this regard, further consideration must be given to the potential negative consequences associated with notifiable HPAI and the risk of false-alarms (Savill et al., 2008) that may be perceived by individual farmers. Ultimately, when developing a risk profile different aspects of risk – initial incursion vs. the potential for spread between farms, as well as the scale of observation – each have different implications for implementing control measures.

## Conclusions

We have shown how particular assumptions for within-farm dynamics result in counter-intuitive implications for pathogen spread at the population-level; increased transmissibility at the within-farm level sometimes presents a reduced transmission risk to other farms. This is the first demonstration of the potential importance of these cross-scale interactions for HPAI in a commercial poultry industry. Together with consideration to the relative importance of network connectivity, these findings have implications for the targeting of HPAI control measures at the farm-level.

## Figures and Tables

**Table 1:** Default parameter values for the within-flock highly pathogenic avian influenza transmission model.

Parameter	Description	Default values/ranges	References
*N*	Mean farm-level flock size (ratio of number of poultry to number of poultry houses)	4500–45,600 birds	PND and CCD^a^
$\bar{n}$	Median flock size across all farms (i.e. across all *N*)	21,500	PND and CCD^a^
*β*_*a*_	Transmission rate via aerosol (h^−1^)	0.01–10 (incrementing by 0.5)	Bouma et al. (2009), Spekreijse et al. (2011b), Tiensin et al. (2007), van der Goot et al. (2005)
*β*_*f*_	Transmission rate via dispersal of infectious faeces (h^−1^)	0.01–10 (incrementing by 0.5)
*β*	Total infection pressure (h^−1^)	*β*_*a*_ + *β*_*f*_
*δ*	Rate of infectiousness onset (h^−1^)	0.021	Bouma et al. (2009)
*γ*	Highly pathogenic avian influenza induced mortality rate (h^−1^)	0.01	Tian et al. (2005), van der Goot et al. (2003, 2005), Webster et al. (2006)
*ε*	Rate of excretion of faecal material in grams (h^−1^)	1	–
*σ*	Rate of decay of infectious faecal material (h^−1^)	0.05	Shortridge et al. (1998)

a PND, Poultry Network Database; CCD, Catching Company Database.

**Table 2:** Summary of catching-team on-farm visit factors (*n* = 108 farms).

Variable	Description	Median value (range) or distribution
Number of poultry	Total number of birds held per farm – used as a proxy for overall farm size	110,963 birds (12,270–384,000 birds)
Number of houses	Total number of poultry houses per farm	6 houses (1–14 houses)
Average flock size	Ratio of total number of poultry to total number of houses per farm	21,583 birds (4571–45,667 birds)
Total number of catching days	Total number of days with catching-team visits over entire Catching Company Data per farm	71 days (9–257 days)
Mean time between catching days^a^	Mean number of days between consecutive catching-team visit days per farm	12 days (4–92 days)
Mean daily caught birds	The total number of birds caught per day, averaged across all individual catching days, per farm	2,055,393 birds (18,982–11,740,723 birds)
Mean daily vehicle loads	The total number of slaughterhouse vehicle loads per day, averaged across all individual catching days, per farm	354 loads (24–2048 loads)
Between-farm association frequency	A measure of farm-level network connectivity, calculated as the total number of associations in a between-farm matrix of associations via slaughterhouses, catching companies or company integration	344 farm associations (105–1453 farm associations)
Integration	Binary categorisation of whether a farm is associated with an integrated company	66% = integrated^b^
Regional location	Categorisation of farms based on their regional locations: North and West of England and Wales	9.26–75.9%^c^
*TR*^*FD*^	Mean farm-level relative transmission risks across all ‘incursion day’ iterations, assuming frequency-dependent transmission	3.69 (0.00002–22.30)
*TR*^*DD*^	Mean farm-level relative transmission risks across all ‘incursion day’ iterations, assuming density-dependent transmission	2.44 (0.00001–23.18)
${\bar{TR}}_{i}^{FD}$	Farm-level relative transmission risk assuming frequency-dependent transmission for a mid-range transmissibility scenario corresponding to *β*_*a*_ ~ 5 and *β*_*f*_ ~ 5	4.67 (0.017 0–18.26)
${\bar{TR}}_{i}^{DD}$	Farm-level relative transmission risk assuming density-dependent transmission for a mid-range transmissibility scenario corresponding to *β*_*a*_ ~ 5 and *β*_*f*_ ~ 5	2.29 (0.004–14.52)

a The median dispersal index (variance-to-mean ratio) for the farm-level time-interval between consecutive visit days was 26 days (range: 11–348 days).

b Based on 100 records for which integration status was known.

c 75.9%, 12.96% and 9.26% of farms were located within the North and West of England and Wales, respectively; note that results for the East of England were excluded due to an extremely small sample size (1.85% of farms).

**Table 3a:** Multivariable linear regression: effect of farm-level factors on relative transmission risk computed for *frequency-dependent* within-flock transmission, ${\bar{TR}}_{i}^{FD}$ (*n* = 98^f^ farms).

Predictor variables^a^	Linear model coefficient^b^	95% confidence intervals	*p*-Value
Intercept^c^	2.114	1.989 to 2.239	<0.0001
Mean daily vehicle loads	**0.002**	**0.0016 to 0.0026**	<**0.0001**
Time between consecutive visit days	−0.033	−0.054 to −0.013	0.002
Network connectivity^d^	0.0004	−5.26 × 10^−5^ to 0.001	0.077
Wales vs. North^e^	−0.203	−0.564 to 0.157	0.266
West vs. North^e^	0.096	−0.214 to 0.405	0.541

**Table 3b:** Multivariable linear regression: effect of farm-level factors on relative transmission risk computed for *density-dependent* within-flock transmission, ${\bar{TR}}_{i}^{DD}$ (*n* = 96^f^ farms).

Predictor variables^a^	Linear model coefficient^b^	95% confidence intervals	*p*-Value
Intercept^c^	1.792	1.655 to 1.929	<0.0001
Mean daily vehicle loads	0.002	0.0014 to 0.003	<0.0001
Flock size	−3.8 × 10^−5^	−5.03 × 10^−5^ to −2.53 × 10^−5^	<0.0001
Time between consecutive visit days	**−0.033**	**−0.0575 to −0.0087**	**0.008**
Network connectivity^d^	0.0004	−0.0002 to 0.001	0.212
Mean daily vehicle loads × flock size	−1.2 × 10^−7^	−1.7 × 10^−7^ to −7.09 × 10^−8^	<0.0001

a See Table 2 for variable definitions and value ranges.

b Results obtained for square root transformed values of ${\bar{TR}}_{i}$.

c Numeric variables were centred and therefore the intercept corresponds to their average values.

d Between-farm association frequency was estimated as a measure of between-farm network connectivity.

e West and North refer to geographical regions of England. Predictors in bold had the largest possible range of effect sizes across all farms – see Supplementary Material Section 6.

f The most influential data points (based on their Cook's statistic) were identified and removed from these analyses (*n* = 2 and *n* = 4 data points were removed from FD and DD analyses, respectively).
