# Supplementary material for: Implications of within-farm transmission for network dynamics: Consequences for the spread of avian influenza
Source: Epidemics. 2013 Jun;5(2):67–76. doi: 10.1016/j.epidem.2013.03.001 (PMC3694308; doi:10.1016/j.epidem.2013.03.001)
Supplement: Supplementary file 1 [file mmc1.pdf]

Supplementary Material:

Implications of within-farm transmission for network dynamics: consequences for the spread of avian influenza

Sema Nickbakhsh[1§], Louise Matthews[1], Jennifer E. Dent[2], Giles T. Innocent[3], Mark E. Arnold[4], Stuart W. J. Reid[5], Rowland R. Kao[1]

[1] Institute of Biodiversity, Animal Health and Comparative Medicine, University of Glasgow, Bearsden Road, Scotland, G61 1QH, UK.

[2] Population Health Research Centre, Division of Population Health Sciences and Education, St. George's University of London, Cranmer Terrace, London, SW17 0RE, UK.

[3] Biomathematics & Statistics Scotland (BioSS), The King's Buildings, Edinburgh, EH9 3JZ, UK.

[4] AHVLA Sutton Bonington, The Elms, College Road, Sutton Bonington, Loughborough, LE12 5RB, UK.

[5] Royal Veterinary College, University of London, Hawkshead Lane, North Mimms, Hatfield, Hertforshire, AL9 7TA, UK

[§]Corresponding author

Email address: sema.nickbakhsh@glasgow.ac.uk

1. Sensitivity to within-flock R_0

For the within-flock highly pathogenic avian influenza (HPAI) transmission model, a range of R_0 values were considered due to the widely varying values reported by the experimental and modelling literature. Two sets of models were generated representing small (~1-4) and large (~3-38) R_0 ranges, which corresponded to average time-to-detections of ~15 days and ~2 days respectively (see Table S1). Importantly, despite these scenarios representing different outbreak characteristics, they gave qualitatively similar results for assessing the effect of flock-level factors on the relative transmission risk (*TR*).

The 'larger' range (R_0~3-38) was selected as it was considered likely that the progression of infection within a flock would be rapid for HPAI (Yoon et al., 2005) whilst also recognising that delay to notification beyond one week was possible (Bos et al., 2007). Exploring a range of transmissibilities, rather than a fixed value, was the fundamental point for these analyses.

Table S1. R_0 range scenarios

Varied parameters	R_0 range ~1-4	R_0 range ~3-38
T_d	8-82 days (median ~15 days)	2-10 days (median ~2 days)
β_a/ β_f	0.004-0.1 /bird/hour	0.01-10/bird/hour
ε	0.1 units/hour	1 unit/hour
**Fixed parameters**		
δ	1/48 hours (2 day latent period)	
λ	1/96 hours (4 day infectious period)	
σ	0.05 units per hour	

T_d = time-to-detection of outbreak; β_a = airborne-mediated transmission rate; β_f =faecal-mediated transmission rate; δ = rate of infectiousness onset; λ = rate of mortality from highly pathogenic avian influenza; σ = rate of decay of infectious faeces.

2. Description of Catching Company Database

The catching Company Database (CCD) represented one major company located in England which serviced predominantly broiler chicken farms; 91% of the farms produced broilers, based on the 46 for which poultry species could be determined. These data consisted of 44,758 individual catching team visits recorded on an hourly basis across a total of 950 days (between January 2005 and August 2007), representing 68 catching teams visiting a total of 415 farms. The CCD captured approximately 2% of the entire British poultry flock (estimated as ~ 24,000 commercial poultry farms from a 2009 extract of the Great Britain Poultry Register). However, these data were estimated to account for 30%-50% of all premises serviced by major catching companies in England and Wales (Dent et al., 2011).

For 181 farms, the total number of catching team visits was considered too few; these farms had fewer than eight recorded catching-team visits in total (the range across the entire 415 farms = 1-369 visits, median = 21 visits). Figure S1 shows the correlation between farm-level mean time intervals between consecutive visit days, and the full data length (i.e. time period over which all visits were recorded). For the farms with fewer than eight recorded visits, these factors were correlated (red points). As it was not known whether the increased time between visit days was an artefact of the limited number of data points, these data were excluded from subsequent analyses. Farms whose flock size could not be determined (and three with extreme values) were also excluded (n=126), leaving 108 farms for these analyses.

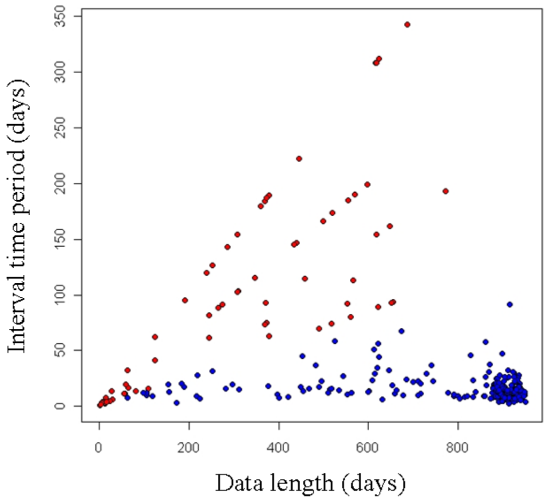

Figure S1. Identifying outliers based on inconsistent catching data. Red points = 181 farms with less than eight recorded catching-team visits. Blue points = 234 farms considered to have a consistent frequency of catching-team visits across the entire time-period of the data.

3. Model simulations: incursion day scenarios

Relative transmission rates (*TRs*) were explored for two scenarios in order to examine the effect of farm-level heterogeneity in the pattern of catching-team visits. The temporally explicit data describing on-farm catching visits was expanded into a matrix of consecutive days, where 1 corresponded to a catching-team visit, and 0 corresponded to no catching-team visit. Each catching day was weighted by the total number of slaughterhouse vehicle loads, φ, across all visits within a single day per farm.

Figure S2 illustrates these scenarios: by varying the assumed incursion day, the impact of the farm-level catching-team visit patterns was determined. Both solid and dashed boxes represent the *incursion via any source* scenario, which allowed incursions to occur via any route (such as through contact with wild birds, the environment, or catching-team visits). In contrast, solid boxes represent the *incursion via catching-team* scenario, which limited the potential for the exposure of people, vehicles and equipment by highly pathogenic avian influenza (HPAI) to the days subsequent to an on-to-farm catching-team visit. The number of incursion day iterations varied per farm and per scenario. For both scenarios, each consecutive day subsequent to the incursion day was matched to the corresponding day of the within-flock outbreak.

Farm (i)	t_1	t_2	t_3	t_4	t_5	t_6	t_7	t_8	…t_{884}
i_1	6	0	0	0	0	0	0	0	0
i_2	12	3	0	0	0	0	0	0	0
i_3	12	0	0	0	3	3	0	0	0
i_4	4	0	0	0	0	0	1	4	0
i_5	1	10	0	0	0	0	4	1	8
i_6	1	0	0	0	0	0	0	0	0
i_7	7	0	0	0	0	0	4	1	0
i_8	3	0	0	6	4	0	0	0	6
i_9	6	0	0	0	0	0	3	7	0
i_{10}	8	0	0	0	0	17	19	0	5
… i_{108}	8	5	0	5	0	0	0	0	

Figure S2. Catching-team visit patterns: an example highlighting the incursion scenarios. *i* = farm; *t* = day; solid + dashed boxes = all consecutive days included in the incursion day iterations for *incursion via any source* scenario for farm 8, including days for which no catching-team visits occurred (i.e. incursion assumed to occur via either catching-team visit or non catching-related mechanisms); solid boxes = catching days included in the incursion day iterations for the *incursion via catching-team* visit scenario (i.e. incursion assumed to occur via catching-team visits only).

4. Sensitivity of *TR* to mortality threshold and flock size

For frequency-dependent (FD) transmission characteristics, an increase to flock size increased the relative transmission risk, *TR* (Figure S3a, moving down grid from top to bottom). Furthermore, increases to the mortality threshold (MT) caused the upper *TR* values to span an increasingly greater β range (Figure S4a, movement across grid from left to right).

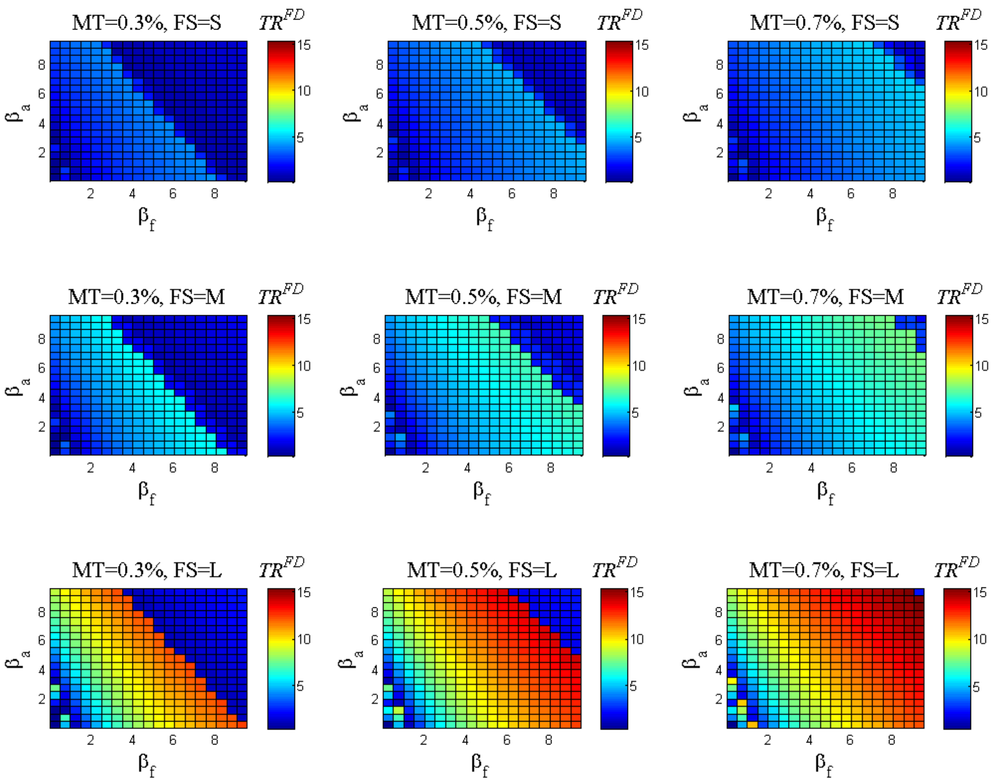

Figure S3a. The relative transmission risks (*TRs*) assuming *frequency-dependent* transmission and incursion via catching-teams. For pairwise combinations of the transmission rate parameters β_a and β_f for increases to farm-level mean flock size (FS) from left to right: small (S; ~4,500-25,000 birds), medium (M; ~25,000-35,000 birds) and large (L; ~35,000-45,600 birds), and for Increases to mortality thresholds (MT) from top to bottom; at least 0.3%, 0.5% and 0.7% mortality of the of initial flock size observed for two consecutive days. All other parameter values set to default (see Table 1 of main text).

However, for density-dependent (DD) transmission, although *TR* scaled with flock size for low-mid-range transmission rates (i.e. $\beta_a + \beta_f$), for mid-to-high range transmission rates (i.e. $\beta_a + \beta_f > 10$) earlier disease detection caused *TR* to peak at increasingly lower transmission rates for increases to flock size (see Figure S3b).

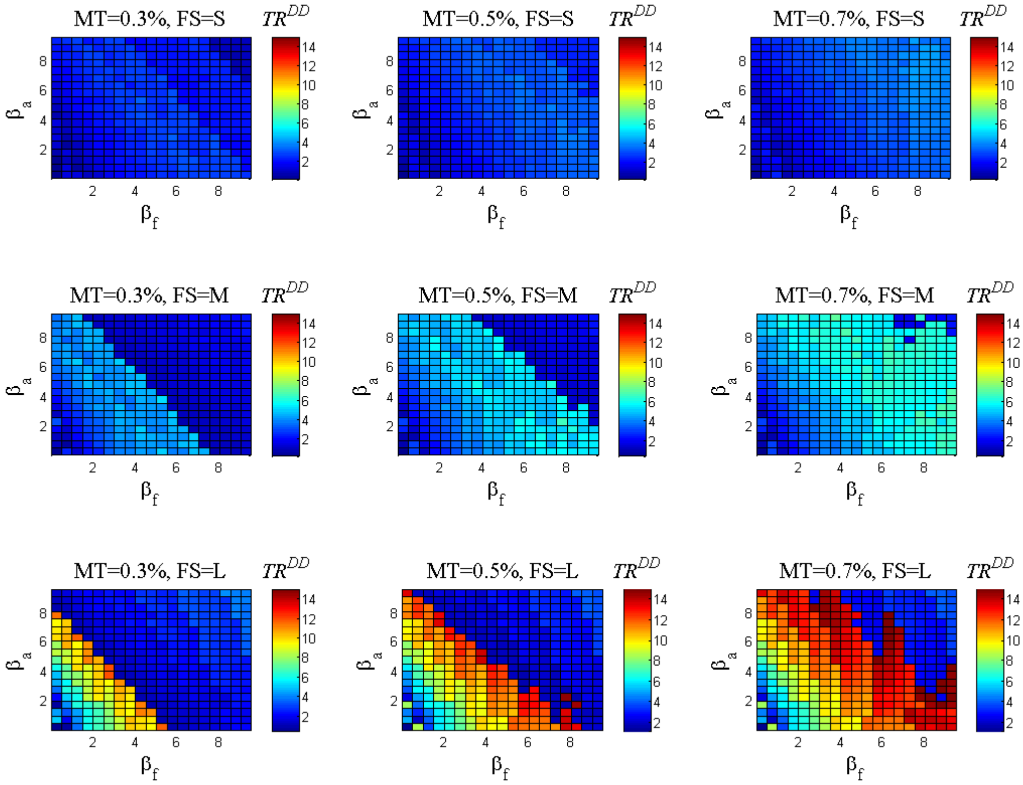

Figure S3b. The relative transmission risks (*TRs*) assuming *density-dependent* transmission and incursion via catching-teams. For pairwise combinations of the transmission rate parameters β_a and β_f for increases to farm-level mean flock size (FS) from left to right: small (S; ~4,500-25,000 birds), medium (M; ~25,000-35,000 birds) and large (L; ~35,000-45,600 birds), and for Increases to mortality thresholds (MT) from top to bottom; at least 0.3%, 0.5% and 0.7% mortality of the of initial flock size observed for two consecutive days. All other parameter values set to default (see Table 1 of main text).

5. Multivariable linear regression: model diagnostics

The aim of the multilevel linear regression analyses was to identify factors that have the greatest effect on the risk of transmission (*TR*) from a farm. A general linear model was used to compare the model inputs with the outcome: *TR* computed for frequency-dependent (FD) and density-dependent (DD) transmission scenarios. In order to remove the effect of uncertainty in transmission parameters these were set at a mid-range value (β_a~5 and β_f~5). Rather than producing a non-linear analysis that could, in principle, fit the data perfectly, we were interested in identifying the main drivers for transmission risk at the flock level.

The final models (see Tables 3a and 3b of main text) displayed no substantial violation to homoscedasticity, normality of model residual distributions (Figure S4) or to goodness of fit (R^2=0.77 and 0.66 for models corresponding to $\overline{TR}$ computed under FD and DD transmission scenarios, respectively). Assessment of the most influential data points (identified by their Cook's statistic) revealed farms that had a significant impact on the model coefficients and their significance-levels (n=2 and n=4 farms for the analysis of $\overline{TR}$ computed for FD and DD scenarios, respectively). These farms were excluded from fitting of the final models presented.

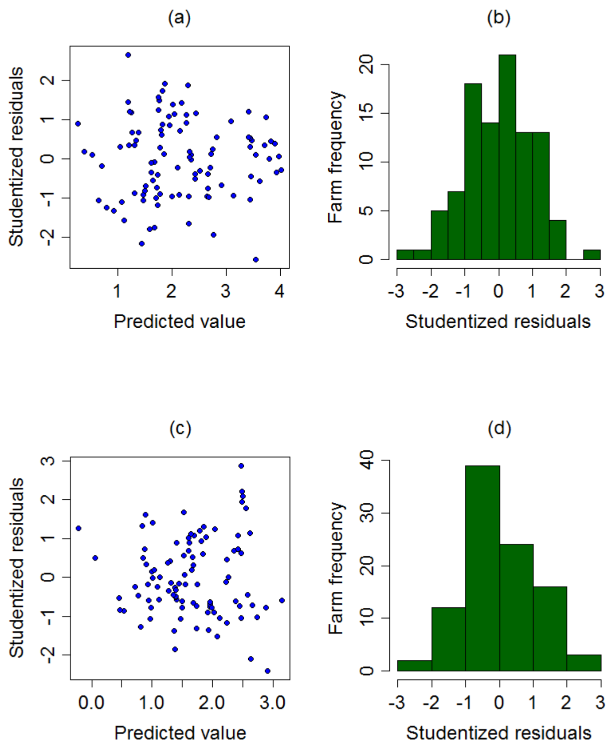

Figure S4. Multivariable linear regression diagnostics for final models. (a,b) For models corresponding to the relative $\overline{TR}$ computed for frequency-dependent (n=98 farms) and (c,d) density-dependent (n=96 farms), scenarios. $\overline{TRs}$ were square-root transformed for the purpose of these analyses.

6. Multivariable linear regression: effect size distributions

The effect of varying a single covariate or factor across the range observed within the CCD dataset was computed. Using this method, importance in model fit (through the model selection process) size of effect and variability between flocks were combined. When considering the range of possible *effect size* across all farms for each predictor, "mean daily vehicle loads" had the potential to have the largest impact on $\overline{TR}$ under FD transmission (Figure S5).

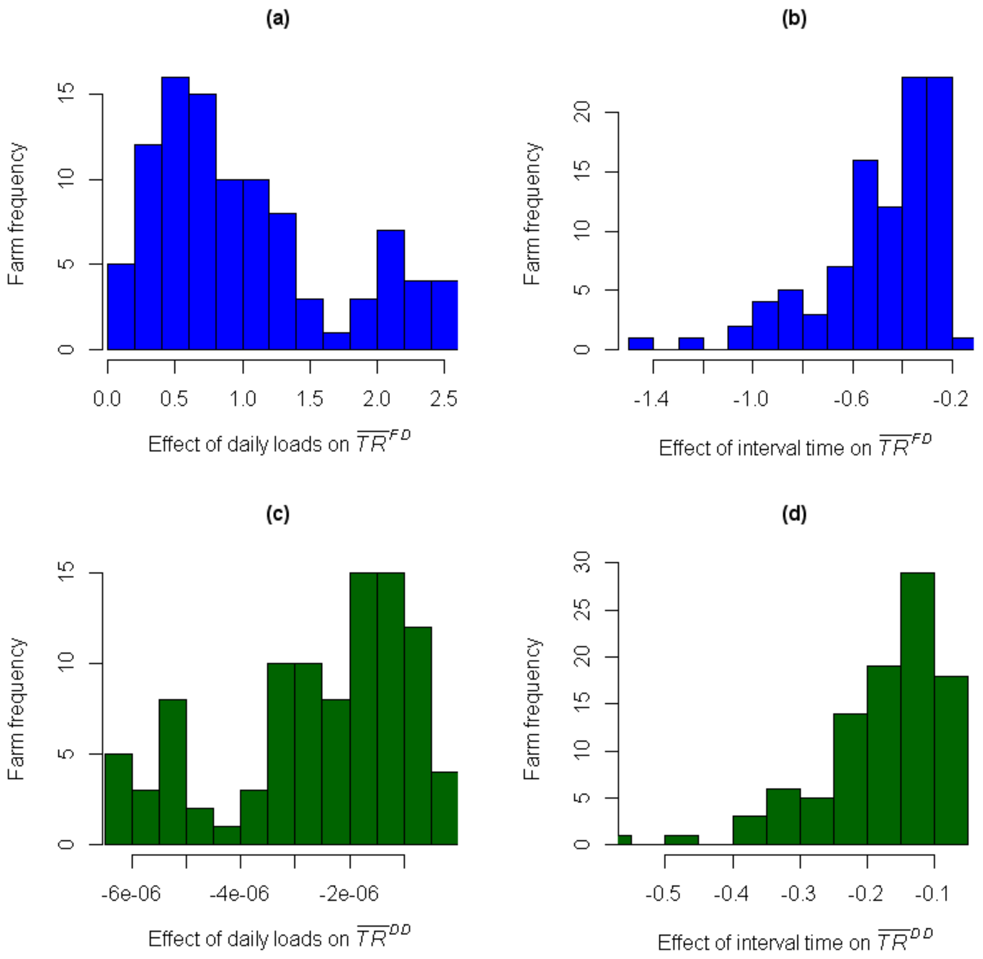

Figure S5. Distribution of effect sizes calculated by multivariable linear regression models. (a,c) Distribution for the effect of mean daily number of vehicle loads for frequency-dependent ($\overline{TR}^{FD}$) and density-dependent ($\overline{TR}^{DD}$) transmission, and (b,d) time-interval between consecutive catching-team visits for $\overline{TR}^{FD}$ and $\overline{TR}^{DD}$ transmission, for the relative transmission potential computed using a mid-range transmissibility scenario. Note that the effect sizes relating to "interval time" are negative due to the decrease in $\overline{TR}$ with increases to the time-between consecutive catching-team visits (see Tables 3a and 3b of main text).

7. References

Bos, M.E., Van Boven, M., Nielen, M., Bouma, A., Elbers, A.R., Nodelijk, G., Koch, G., Stegeman, A., De Jong, M.C., 2007. Estimating the day of highly pathogenic avian influenza (H7N7) virus introduction into a poultry flock based on mortality data. Vet Res 38, 493-504.

Dent, J.E., Kiss, I.Z., Kao, R.R., Arnold, M., 2011. The potential spread of highly pathogenic avian influenza virus via dynamic contacts between poultry premises in Great Britain. BMC Vet Res 7, 59.

Yoon, H., Park, C.K., Nam, H.M., Wee, S.H., 2005. Virus spread pattern within infected chicken farms using regression model: the 2003-2004 HPAI epidemic in the Republic of Korea. J Vet Med B Infect Dis Vet Public Health 52, 428-431.
